# The Good, The Bad and The Ugly: Disaster Risk Reduction (DRR) Versus Disaster Risk Creation (DRC)

**DOI:** 10.1371/4f8d4eaec6af8

**Published:** 2012-06-21

**Authors:** James Lewis, Ilan Kelman

## Abstract

In understanding and trying to reduce the risk from disasters, connections are often articulated amongst poverty, vulnerability, risk, and disasters. These are welcome steps, but the approach taken in top-down international documents is rarely to articulate explicitly that vulnerability accrues from a wide variety of dynamic and long-term processes. Neglecting these processes—and failing to explore their links with poverty, risk, and disasters—tends to encourage disaster risk creation. This paper identifies seven examples of on-the-ground realities of long-term vulnerability within two clusters: Endangerment: 1 Environmental degradation. 2 Discrimination. 3 Displacement. Impoverishment: 4 Self-seeking public expenditure. 5 Denial of access to resources. 6 Corruption. 7 Siphoning of public money. Examples are presented as vignettes, many contemporary and many rooted in historical contexts, to demonstrate the extent to which “vulnerability drivers” emanate from greed, the misuse of political and commercial power, mismanagement and incompetence amongst other behaviours. Moving forward to the tackling of disaster risk creation, instead of simply seeking disaster risk reduction, requires detailed investigation into these contemporary and historical realities of the causes of vulnerability. That would support the integration of disaster risk reduction within the many wider contexts that foment and perpetuate vulnerability.

## Note regarding the title

“The Good, the Bad and the Ugly” (1966) directed by Sergio Leone and starring Clint Eastwood, Eli Wallach and Lee Van Cleef is billed as “A bounty hunting scam joins two men in an uneasy alliance against a third in a race to find a fortune in gold buried in a remote cemetery.” http://www.imdb.com/title/tt0060196/

## Introduction

Bronowski’s (1973, p. 15)^1^ view “There cannot be a philosophy, there cannot even be a decent science, without humanity…For me, the understanding of nature has as its goal the understanding of human nature, and of the human condition within nature.” is poignant in contemporary interpretations of disaster risk: “if most disaster management agencies and governments ignore the social and political origin of disasters, how can disaster risk reduction ever happen?” (Heijmans, 2001, p. 4)^2^ . Writing about the 1997-1998 forest fires in Indonesia, Heijmans (2001, p. 3)^2^ interpreting from Harwell (2000)^3^ describes how conflict between local farmers and adjacent plantation owners inhibited the response, with farmers believing that the fires were set to drive them out so that the plantations could take over:

It is not poverty or the ‘slash and burn’ practices of poor farmers that set the degradation of nature in motion, but the greedy and unjust behaviour of concessionaires, politicians, and law enforcement officers involved in the conversion of forests to plantations. This created the vulnerable ecological and social conditions for the fire disaster.

Crucial linkages amongst poverty, vulnerability, and disasters are reiterated similarly for UNISDR/SOPAC’s (2009)^4^ investigation of the relationship between disasters and poverty in Fiji:

The Fiji case study confirms that a two-way relationship exists between natural disasters and poverty, and suggests that initiatives to reduce disaster risks can also help reduce poverty. Further, it also confirms that efforts to reduce income poverty and other basic social conditions can help reduce people’s vulnerability to disaster. That is, any poverty reduction strategy should also account for the DRR issues, for synergy in reducing both disaster impacts and poverty.

So far, so good, but it has taken a very long time to have this short conceptual distance engrained in international documents. UNISDR (2009)^5^, for instance, is a major step towards disaster reduction (DRR) and is a significant leap from the comparatively platitudinous 2005 *Hyogo Framework for Action *(HFA; UNISDR, 2005)^6^ *, *as part of the final report of the Hyogo World Conference on Disaster Reduction of the same year. This, notwithstanding that those same linkages were forged in the early 1970s (e.g. O’Keefe et al., 1976)^7^ and, frequently, afterwards (e.g. Hewitt, 1983)^8^.

Profusely mapped and tabulated, the global, national, and local impacts of disasters are described as exacerbated by “‘risk drivers’ such as increasing urbanisation, poor urban governance, vulnerable rural livelihoods and the decline of ecosystems” (UNISDR, 2009, p. iii)^5^. Stated as leading to “massive human misery and crippling economic losses” (UNISDR, 2009, p. iii)^5^, the political origins of poverty and of vulnerability to disasters, however, receive no fully direct or explicit mention and neither does “greedy and unjust behaviour of concessionaires, politicians, and law enforcement officers” (Harwell, 2000, p. 330)^3^. Limited articulation is given for such realities or the depth of those realities. Information supplied has to do with *what,* *where* and *how big* disasters have been, rather than focusing on *why* or to *whom.* The realities of the *status quo* tend to remain unexplored, obscure, and insufficiently penetrated.

Specification of these four risk drivers could potentially be taken to assume that it is not necessary to look further—or, to the extreme, that vulnerability happens in no other contexts, in no other ways, in no other places, or with no other “drivers” of its own. That is not the case (e.g. Harwell, 2000; Hewitt, 1983, 1997; Lewis, 1999; Wisner et al., 2004)^3^ ^8^ ^9^ ^10^ ^11^. Vulnerability accrues by its own “drivers” and by its own volition, not always contained by the four named risk drivers, especially through a wide variety of dynamic and often long-term processes (Lewis, 1987)^12^, the neglect of which encourages disaster risk creation (DRC).

How much is disregard of this interpretation of vulnerability, as well as equal disregard for its behavioural causes as cited in this paper, the reason why disaster risk reduction (DRR) is not being achieved at the desired rate? A survey involving 7,000 people in 48 countries (GNCSODR, 2009)^13^ indicated “a significant gap between national and local level action” (p. iv) and that progress at the present rate “will not deliver the required reduction in disaster losses by 2015” (p. 12). Consequently, at its current pace of implementing change, the HFA cannot reach its stated goals, especially Priority Action 4 “Reduce the underlying risk factors”.

Interpreting “The Good, the Bad and the Ugly” in its three categories, this paper identifies specific and pragmatic examples of on-the-ground realities of vulnerability for comparison with wide-scale, remote, and relatively top-down conceptions within necessarily internationally agreed and produced documents. The examples are presented as vignettes, illustrating that “vulnerability drivers” often emanate from greed and the misuse of political and commercial power. That occurs in all contexts, not just in the four named risk drivers. It is within these behavioural climates that DRC festers.

Here, “the good” is interpreted as positive DRR measures, but expounding on some of the problems with the presentation of what is taken to be “good”. Meanwhile, “the bad” and “the ugly” are interpreted as processes that increase, or fail to decrease, vulnerability and that actively or inertially stymie or constrain DRR efforts. “Bad” is effectively malfeasance—actions in which the perpetrators to a large degree understand, or should understand, that their specific choices, over which they have power, are causing disaster-related problems, usually (although not always) for others. “Ugly” does not refer to “ugly in appearance”, instead implying “ugly in intent and action” even though there might be limited understanding of the vulnerability- and disaster-related implications, not out of wilful ignorance but due to realities, especially externally imposed realities, that often preclude integration of disaster implications into decision-making processes.

As will become clear through the specific case studies, the line between “bad” and “ugly” is often blurred and the literature has ongoing debates regarding whether or not such activities should be separated. Cannon (2008)^14^, for instance, argued that some disasters and vulnerability are “innocent” because sometimes people make active choices—perhaps through group behaviour, religious beliefs, or priorities other than disaster risk reduction—to accept vulnerabilities and risks for livelihood or quality of life purposes. That is fair as a truism, corroborated through ample field evidence as well as our own day-to-day decisions as disaster professionals, but the moniker “innocent” is unconvincing and introduces the possibility of external pressures, or layers of ethical judgements, which ought to be further explored.

If someone with full knowledge of flood risk settles in a floodplain, thereby encouraging others to do so even though the others do not have full knowledge of the flood risk, how “innocent” is that? If people make livelihood choices to live on the slopes of a frequently erupting volcano, are we certain that social processes and power structures did not preclude other options? How do we balance the vulnerabilities and risks of cultivating fertile, landslide-prone hillsides in comparison to land which is less prone to landslides but more prone to rainfall-related drought? More importantly, what social processes or underlying factors led to those two choices (or outmigration) as being the only options available?

Many of the answers to these questions, and the separation of “bad” and “ugly”, could relate to who and which processes provide the options available and who or which processes take the final decision within the bounded frame of only certain options being available. A single paper cannot delve into that for the numerous realities of vulnerability presented here. For discussion purposes, we separate “the bad” and “the ugly”, recognising that, at deeper levels of the case studies, they are likely to be heavily intertwined—and even inexorably linked to “the good”. As per the final example in the previous paragraph, the decision to farm landslide-prone hills can be a drought risk reduction strategy while the decision to farm drought-prone plains can be a landslide risk reduction strategy. The good, the bad, and the ugly are inexorably linked—and it is not even clear that “good” means the minimum risk feasible (e.g. Kelman and Mather, 2008)^15^.

## The good – as far as it goes

Internationally produced United Nations documents reflect the valid concerns of that organisation and of its member governments. They also embody the characteristic disadvantage that governments do not welcome exposure of their own shortcomings. How else could governments be expected to commit themselves to signing up to report content unless there are no indictments, no fault-finding, no blame, and no negatives reflecting on themselves?

Consequently, entire initiatives, such as the World Conference on Disaster Reduction, the HFA, and the Global Assessment Reports, are perpetually required to trade on well-meaning innate optimism, positive drive, and honourable common good for the future of humankind. What such documents do not express, therefore, is that governments, societies, and communities, whether rural or urban, include the bad and the ugly as well as the good, the corrupt as well as the ethical, and the inert and the weak as well as the powerfully active.

Such processes, and these documents, reflect and are the product of an assumed “equality of the good” that cannot represent or express society’s inequalities and injustices that are themselves a principal cause of vulnerability. In part expressed as “poverty”, causes of poverty are not always entirely recognised or considered – except that of disasters themselves. As this paper demonstrates through the lens of vulnerability to disasters, poverty is not simply a static, immutable and inconvenient fact. Instead, it is the by-product of actions and inactions across economic and governance sectors. That it may be rare for these sectors to be represented within the DRR mandate does not make them irrelevant for DRR. Conversely, it becomes crucial for DRR issues to be represented, comprehended and implemented integrally within the management of each of those other sectors, directly placing DRR work within and across other development activities.

But even if that were achieved, another factor emerges: DRR appears to not always acknowledge that societies inevitably and invariably include those with less admirable motives (Cannon, 2007)^16^. Greed and self-indulgence serving to exacerbate inequality (Dorling, 2010)^17^, and other people’s vulnerability, is an unfortunate reality that prevails beyond most concepts of governance, operating powerfully and pervasively *inter alia* contrary to the objectives of DRR at domestic, group, corporate and institutional levels. Good governance applies to the rural as well as to the urban, to the rich as well as the poor. It cannot be restricted to initiating or permitting favourable systems and procedures but has to prevent and inhibit other procedures that, whilst advantageous to the powerful few, result in widespread deprivation for the many.

Whilst exclusion of the negative is accepted practice for internationally produced documents, it will continue as necessary to acknowledge internationally investigative journalism (Bureau of Investigative Journalism, 2011)^18^, media exposures of biased reportage, and socially conscientious revelatory “leaks”, in order to achieve the needed greater awareness of prevailing political and social realities. These courageous sources serve usefully to temper fairy-tale fantasies as they take their place alongside the acceptably comfortable but complacent, internationally conceived, jointly contributed, and governmentally signed documents – along with some guidelines and academic texts.

Disasters for vulnerable people are often the consequence of longer-term negative pasts created by others, against which concepts of personal and community resilience have sometimes been powerless (e.g. Hewitt, 1983, 1997; Lewis, 1999; Wisner et al., 2004, 2012)^8^ ^9^ ^10^ ^11^ ^19^. DRR needs to recognise these processes of “epidemiological” vulnerability now, so that disasters of the future might realistically be reduced: “Disaster reduction must maintain temporal awareness of pasts and futures, as well as of the present, so that as vulnerability processes come to be recognised, conditions and consequences are reduced. Meanwhile, resilience fiddles on the re-arranged deckchairs while Rome burns.” (Lewis and Kelman, 2010, p. 208)^20^.

As it is, regardless of prevailing realities, “DRR” has become the universal acronym to mean whatever interpretation is chosen for it, no matter what the intentions were of its origins. The *Global Assessment Report* (UNISDR, 2009)^5^ will not change that, although by its content, DRR has now been made a shade more accurate. Nevertheless, the invidious processes continue to prevail from which ensue vulnerabilities and the origins of DRC, unrecognised and unaccepted by many, and being mostly unhindered and unchecked.

## Vulnerability drivers: the bad and the ugly

People’s vulnerability is caused by many actions and activities, mostly perpetrated by others (Hewitt, 1983, 1997; Lewis, 1999; Wisner et al., 2004)^8^ ^9^ ^10^ ^11^. Behaviour by a few politically or commercially powerful frequently causes endangerment to or impoverishment of many others, as is illustrated by seven categories incorporating bad and ugly behaviours and consequent actions:

- Endangerment:

1. Environmental degradation.

2. Discrimination.

3. Displacement.

- Impoverishment:

4. Self-seeking public expenditure.

5. Denial of access to resources.

6. Corruption.

7. Siphoning of public money.

These behaviours are identified as sources of actions creating conditions of endangerment and impoverishment leading to vulnerability. But though these behaviours lead collectively to vulnerability, the action of each is brought about by separate and distinctly different reasoning, even within each category. For instance, marginalisation has long been identified as a source of vulnerability, but behaviours that are the sources and causes of the elements of marginalisation, internally and externally to a community, need to be recognised. That means that, in the recent and historical case studies (Table 1), some categories are distinctly “bad”, some are distinctly “ugly”, and some include examples of both, separately or in combination.

In the face of the contexts, circumstances, and complexities of the reality of the cases, and often the entanglement of “the bad” and “the ugly”, is the world-wide institutionalisation of DRR serving more as cover-up, papering over the cracks of a façade? Does the achievement of top-down institutionalised DRR mean that thought and enquiry are no longer desired? More benevolently, perhaps, is it that the right hand is continuing to do one thing whilst the left hand does another (Lewis, 1984, p. 179)^21^, not wishing to fully admit how the causes of vulnerability require attention in order to adequately address DRR? In short, DRR has some way to go before it can be recognised as containing all of the answers required for dealing with vulnerability, risk, and disaster. Meanwhile, DRC proceeds apace.

Examples from around the world have been collected of this consequence arising from bad and ugly behaviours, arranged in the seven categories within the two clusters. Table 1 provides an overview, followed by text describing additional selected examples as vignettes.

## Table 1: Cases of endangerment and impoverishment

**Table:** 

*Country*	*Year*	*Details*	*Source*
*ENDANGERMENT*			
*1. Environmental degradation: bad and ugly.*			
*India*	*2010*	*Over-exploiting environmental resources for development, thereby augmenting environmental hazards.*	*Bidwai, 2010^22^*
*Pakistan*	*2010*	*Illegal logging, increasing flood/landslide hazards plus the felled trees become another hazard when swept away by flood waters.*	*Shamsie, 2010^23^*
*United Kingdom*	*2007*	*Placing housing on river flood plains, often due to greed from developers seeking quick profit and local authorities seeking quick tax revenues.*	*Lewis and Kelman, 2010^20^*
*Japan*	*2005*	*114 new buildings, including 36 hotels, are deemed to be of inadequate construction for the hazard faced.*	*Lewis, 2010^24^*
*Germany*	*2002*	*Placing housing on river flood plains, especially greedy local authorities seeking development.*	*Pierce, 2002^25^*
*2. Discrimination: bad.*			
*Spain*	*2011*	*Sub-standard living conditions for migrants preventing them from enacting DRR.*	*Lawrence, 2011^26^*
*United Kingdom*	*2005*	*Marginalised citizenship which exposes those affected to hazards.*	*Hogan and Marandola, 2005^27^*
*USA*	*2005*	*Disadvantaged living in physically vulnerable areas, exposed by Hurricane Katrina.*	*Laska et al., 2006^28^*
*Tonga*	*1989*	*Financial judgements denied the social need for shelter, leaving the poorest without post-disaster housing and thus made more vulnerable, serving DRC.*	*Lewis, 1989^29^*
*Dominican Republic*	*1982*	*Exclusion from arable land increasing vulnerability to famine.*	*Jeffery, 1982^30^*
South Africa	1960s onwards	Townships as ghettos of discrimination, which places them in hazardous locations and which inhibits local DRR.	UNHabitat, 2010^31^
3. Displacement: bad.In all these cases, moving people from their land and communities increased their vulnerability by resettling them in unfamiliar places without adequate support for DRR in the new location, especially where the people experienced unusual for to them.			
Brazil and Guyana	2011	US$17 billion Amazon dam project displaces tribes.	Reuters AlertNet, 2011^32^
Philippines	2001	One dam displaced 61,700 people.	Heijmans, 2001^2^
China	1959-1989	10 million people displaced by water projects.	Cernea, 1996^33^
India	1956-1996	18.5 million people displaced by dams and mines.	Cernea, 1996^33^
IMPOVERISHMENT			
4. Self-seeking expenditure: ugly.			
South Africa	2009-2010	Doubling of project costs implies misuse of funds which could have been used for DRR or sustainability projects.	Donnelly, 2010^34^
Italy	1945 onwards	Less infrastructure in the south for more money spent.	Golden and Picci, 2005^35^
5. Denial of access to resources: bad and ugly.			
Pakistan	1998	Skewed access to productive land, affecting ability to reduce vulnerability through reliable local food.	Mustafa, 1998^36^
Tuvalu	1941-1945	Long-term depletion of crops due to World War 2 airfields, affecting ability to reduce vulnerability through reliable local food.	Telavi, 1983^37^
Martinique	1635 onwards	Unequal distribution of landholdings, affecting ability to reduce vulnerability through reliable local food.	Jeffery, 1981^38^
6. Corruption: bad.In all these cases, the money could have been used for DRR or vulnerability reduction measures, especially in wider contexts of supporting local livelihoods and aiming for sustainable communities. Where noted, money lost through corruption could have been used to increase the hazard resistance of infrastructure.			
Bangladesh	2011	Anti-graft official accepted US$14,000 bribe.	AFP, 2011^39^
Indonesia	2011	Tax official took bribes of millions of US$ in gold.	Deutsch, 2011^40^
Kenya	2011	Local misuse of development funds in 47 counties.	Daily Nation, 2011^41^
China	2007	Bribes and theft are estimated at 8% of government spending.	Pei, 2007^42^
Turkey	1999	Earthquake damage from corrupt construction control.	Lewis, 2008a^43^
7. Siphoning of public money: ugly.In all these cases, the money could have been used for DRR or vulnerability reduction measures, especially in wider contexts of supporting local livelihoods and aiming for sustainable communities.			
Gabon	2010	US$36 million embezzled.	Ryan, 2010^44^
Sudan	2010	President siphoned US$9 billion overseas.	Hirsch, 2010^45^
Tajikistan	2010	President controls revenues from state industries.	Harding, 2010^46^
India	2008	The country’s illegal economy represents 50% of GDP.	Kar, 2010^47^
Kenya	2001-2005	Government money was paid to a fictitious firm for contracts. Some of that was paid back.	Githongo, 2005^48^
Mexico	2000-2009	Illicit financial outflows of US$46.24 billion.	GFI, 2011a^49^
Angola	1993-2002	US$4.68 billion lost between 1993 and 2002.	Shaxson, 2011^50^
Italy	1945 onwards	Massive siphoning of development funding.	Golden and Picci, 2005^35^

**Endangerment 1: Environmental degradation**

Development affecting the environment may be initiated, perpetrated or permitted that places people at risk when they occupy that development. The risks may arise from environmental degradation or environmental modification that creates or augments hazards.

***India***

Bidwai (2010, online)^22^ describes India’s growth as having two negative sides: “persistent poverty, social bondage and economic servitude” and “sleaze and collusive business-politics relations that mock transparency, accountability, democratic policy-making and the public interest.” He notes that “what counts is privileged access to natural resources and the national commons, most critically land, which is at the core of the government’s discretionary powers”. Amongst other examples are “mining and metallurgical projects that blatantly violate environmental regulations; corporate land grabs in the guise of export promotion zones; the razing of virgin tropical rainforest to make way for opulent housing”. These actions have a tendency of exacerbating, even creating, flood, landslide, and drought hazards.

***Pakistan***

In the floods of 2010, millions of people were affected by torrential monsoon rains and consequent floods. By August, entire villages had been washed away, 1,600 people had died, 2 million had been made homeless, and dissent was leading to political instability (Shah, 2010)^51^. It was reported that “the timber mafia, which has a particularly strong hold on the areas now affected by flooding [is] one of the most powerful and ruthless organisations within Pakistan…engages in illegal logging, which is estimated to be worth billions of rupees each year” (Shamsie, 2010, online; see also Ali et al., 2006)^23^ ^52^. In addition to deforestation contributing to flooding, landslides, and soil erosion, illegally felled trees hidden in ravines were dislodged by floodwaters and destroyed bridges essential for access to flooded areas. Dams and retaining walls intended for flood control were possibly weakened as well which might have contributed to some of the inundation, although detailed investigations have not been carried out (Shamsie, 2010; Sudmeier-Rieux, 2011a)^23^ ^53^.

**Endangerment 2: Discrimination**

Ethnic, religious, gender or (dis)ability-based discrimination, for example, may lead to deprivation, victimisation, or the forced adoption of hazard-prone habitation. Being discriminated against also reduces the options available for individuals and communities to adopt DRR measures.

***Nepal***

****Nripal (2008, online)^54^ writes:

In my studies of a few villages of Nepal, I found that vulnerability is a political issue. Culture is a response to politics. Before we talk about preparedness or coping mechanisms that cultures have adopted in different temporal and spatial settings... it is necessary to address the root causes of vulnerabilities...In the Dhadhing district of Nepal, ethnic groups like Chepangs live in very steep lands...studies have shown that they are very adaptive to those settings. But it is the policies of institutionalized discrimination...over about eight hundred years, that has forced them to live in steep slopes. They have adapted well to those harsh conditions... but that does not mean they are completely safe. Food insecurity and poverty still haunt them and they are the first victims of hazards like landslides. They are living in the edge of vulnerability, not because of their cultural inclination but because of institutionalized discrimination against them.

Sudmeier-Rieux et al. (2011b)^55^ confirmed exactly this situation by comparing two villages in Nepal with landslide risk that is comparable, but with differing livelihoods and economic opportunities.

**South Africa**

**Sectors of underdevelopment, such as South African townships, are ghettos of discrimination and deprivation and are dynamos of concentrated vulnerability. Most are overcrowded and prone to disease, fire, storms and flooding while the inhabitants experience forced removal and displacement without alternative accommodation.

UNHabitat (2010, pp. 209-210)^31^ states:

In Durban, Cape Town and Johannesburg, recurring social catastrophes (especially fires and floods devastating entire communities) are viewed by officials as ‘natural disasters’ or faults caused by their low-income victims. Yet, they are clearly a product of asymmetric public policy choices that favour the wealthy at the expense of the poor. The poor are most exposed and vulnerable to disasters and disease...Horrific fires in Alexandra, Johannesburg, are a product of informal settlement layout, overcrowding, highly combustible building materials and inadequate strategies for fire prevention... the most effective prevention of such fires would be provision of affordable electric power to replace the fire hazard of paraffin lamps and candles.

**Endangerment 3: Displacement**

Removal of habitation and people to make land available for other purposes, without compensation or alternative shelter, deprives families of accustomed livelihoods and exacerbates their vulnerability to natural hazards. Forced evictions are reported by Amnesty International (2011)^56^ in places such as Kenya, Nigeria, Paraguay, Serbia and Zimbabwe. When deprived of familiar environments without support for learning about one’s new location, already vulnerable people can be subject to hazards with which they are unfamiliar.

One major cause of displacement is the construction of dams for hydro-electricity, with indigenous people particularly having their livelihoods devastated along with being threatened by extinction (Survival International, 2010; World Commission on Dams, 2000)^57^ ^58^. Examples include Ethiopia, Kenya, Malaysia, Peru, and China. In such cases, vulnerability is increased because the new location is unfamiliar, traditional livelihoods and coping mechanisms have been disrupted and adequate support for long-term DRR is usually not available. These consequences result in the case studies below as well.

**Brazil and Guyana**

Brazil is to construct a series of dams on the River Madeira, one of which would be the third largest in the world, despite protests since the 1980s by Kayapó Indians and other tribes. More large dams are planned for northern Brazil and southern Guyana, including the Upper Mazaruni dam which was stopped after protests, but is likely to be revived (Survival International, 2010)^57^. In January 2011, Brazil’s environment agency approved the commencement of a 240-hectare site clearance project for the Belo Monte dam in the Amazon, with a budget of US$17 billion and displacing numerous native tribes (Reuters AlertNet, 2011)^32^.

**Philippines**

Locally perceived as more disastrous than natural hazards, are governmental ‘development’ projects for dams, irrigation, mining operations, plantations and recreation areas, all requiring conversion of prime agricultural land to industrial and commercial use (see also Gaillard et al., 2007)^59^. Local communities are often not consulted, but are displaced, losing their rights, livelihoods, and lands. Such projects, having immediate negative effects on local poor communities and known as “development aggression” (Heijmans, 2001,p. 5)^2^, are considered as human-made disasters more difficult to cope with than with those of a typhoon. Typhoons are said to destroy crops, houses and infrastructure, but not to undermine the basis of people’s means of survival. Displacement deprives people of their land, the most crucial resource to sustain their livelihood. Government or private compensation is far below the amount needed to rebuild a livelihood elsewhere, and land is not made available (Heijmans, 2001)^2^.

## Impoverishment

Epitomising the state of impoverishment, Washington DC based Global Financial Integrity issued two reports in the early months of 2011. *Illicit Financial Flows from Developing Countries: 2000-2009* (GFI, 2011a)^49^ describes how much of the colossal amounts of money secreted offshore has resulted from the siphoning of funds from developing countries, further impoverishing those countries and social conditions within them, thereby perpetuating and augmenting vulnerability. An additional cause of globally continuing poverty is described in the second report, *Transnational Crime in the Developing World* (GFI, 2011b)^60^, which notes a US$650 billion “global illicit flow of goods, guns, people, and natural resources” (p. v) that functions easily in conditions of underdevelopment, poor governance, and fragile states, conditions that its activities perpetuate and that preclude effective and adequately resourced DRR.

**Impoverishment 4: Self-seeking expenditure of public money**

Self-seeking expenditure manipulates funds, that otherwise could be applied to development, such as DRR work. Instead, these funds are put towards projects that benefit the manipulator or development expenditure is used as a vehicle for personal gain. Either process skews expenditure that could otherwise be applied towards raising the quality of life and reducing vulnerability for wider sectors of society or communities at large.

While no guarantee exists that unsiphoned funds would be used for DRR, the fundamental principle is that public money ought to be used for public good. When that does not happen, self-seeking expenditure is epitomised. By denying the public the benefits from the expenditure of public money, vulnerability is necessarily created. Cases of abuse of entrusted power for personal gain leading to increased community vulnerability are given below as corruption and siphoning of public funds.

**South Africa**

**Police are an inherent part of community resilience and are involved in some DRR activities, as well as in post-disaster response. Where police abuse the system or fail in their duties, vulnerability is augmented, partly because the police are not playing their expected role and partly because the populace will not trust the police to assist.

During 2009 and 2010, three new police stations were constructed at Inanda, Ezakheni and Hebron. Inanda Police Station took six years to build, during which costs soared from a budgeted ZAR15.9 million to an actual cost of ZAR42.0 million. Ezakheni Police Station had an estimated cost of ZAR10.6 million that grew to an actual cost of ZAR23.9 million. Hebron Police Station began construction in June 2006 and was not complete at September 2010 when its budgeted cost of ZAR14.7 million had by then risen to ZAR18.6 million and was still increasing. According to Donnelly (2010, online)^34^, “The average cost of consultants was 18% of a project’s budget, but in some projects it was as high as 25%”. Extended completion times and higher overall payments suggest contractor and consultant complicity.

Overall, for these three police stations at the time of reporting by Donnelly (2010)^34^, ZAR84.5 million of public money had been spent on three contracts budgeted at a total of ZAR41.2 million – an overall expenditure of more than twice the budgeted cost. That misuse of public money draws resources directly away from DRR, and other community development concerns, not just through moving money away from the public good but also through the police apparently being complicit in that process. In this case of trade mispricing, six police stations, or their developmental equivalent, could have been provided for the ultimate actual cost of three (Lewis, 2011b)^61^. That would have contributed to reducing community vulnerability through policing while instilling trust in the police as part of building a resilient community.

**Vietnam**

**Climate change influences resilience to natural hazards, with climate change adaptation being part of DRR. In June 2012, three of four climate change research projects in Vietnam funded by the Danish International Development Agency (DANIDA),were stopped by the Danish Embassy due to fraudulent expenditure by Vietnamese participants. Out of the approximately US$3.45 million in aid for the projects, US$2.45 million had been distributed when US$550,000 was found to have been misused (Talk Vietnam, 2012)^62^. The projects comprised assessments of climate change impacts on land use and livelihoods, socio-economic development, estuarine ecosystems and the effects upon rice production of submergence and salinity. All these activities are part of DRR and link to social improvement, well-being and poverty reduction.

**Impoverishment 5: Denial of access to resources**

Some people’s actions, inadvertent or deliberate, have caused others to be, or to become, deprived of resources that either belonged to them or that were commonly available. Such actions may have been perpetrated in history, but their long-lasting consequences continue to be a part of present-day conditions, having imposed vulnerability, and may have come to be regarded as permanent, normal and acceptable.

**Tuvalu**

**During World War 2 on Funafuti, the main island in what was then the Ellice Islands, and is now Tuvalu (Telavi, 1983, p. 142)^37^:

*In the construction of the airfield a large portion of the land formerly used for growing *pulaka* and *taro* was covered up. The local people were later compensated yet they suffered an enduring loss. *Pulaka* is no longer a staple food, although during the war it did not matter. The American occupation brought them more food - as well as cigarettes, soap and kerosene - more than they had ever had before. Many still think of that as the best time of their lives.*

For the outer island of Nanumea (Telavi, 1983, p. 143)^37^:

*most damage done on the island was done by its defenders. The airfield took up one sixth of the land area, and to make it the Americans destroyed nearly half of the coconut trees, 22,000 out of 54,000. Moreover, efforts to replant that land have not been very successful. The coral is packed too hard for the trees to grow properly*.

Reliable local food is reduced, decreasing self-sufficiency and augmenting vulnerability.

**Impoverishment 6: Corruption**

The link between corruption and disasters is more pervasively invidious than its obvious after-the-fact effect manifesting in, for example, earthquake damage (Ambraseys and Bilham, 2011; Lewis, 2008a; Chien, 2008)^43^ ^63^ ^64^. At one time, corruption was typically condoned as “usefully functional” or as grease for the wheels of bureaucracy, and more often ignored than investigated. That has been the case even though it deprives the poorest of what little money they have - by which poverty is exacerbated and, at higher social levels, it may keep undeserving elites in power (Hoogvelt, 1976)^65^. Now, it is recognised that the hierarchical greed within corruption, its spread and extent, and its scale, consequently distorts the economy, increasing costs of goods and services. As Johnston (2009, online)^66^ notes, “The links between corruption and poverty affect both individuals and businesses, and they run in both directions: poverty invites corruption, while corruption deepens poverty.”

The World Bank’s Stolen Asset Recovery Initiative concludes for developing countries that “The cross-border flow of the global proceeds from criminal activities, corruption, and tax evasion is estimated at between $1 trillion and $1.6 trillion per year” (UN and World Bank, 2007, p. 1)^67^. Using those figures, Baker et al. (2008: online)^68^ comments:

*This dwarfs foreign aid, which totalled about $50 billion a year through the 1990s and is about $100 billion today. So we have $50-100 billion of aid flowing into poor countries, and $500–800 billion of dirty money flowing out. In other words, for every dollar Western governments have been handing out across the top of the table, crooked Western banks, businesses and middlemen of various descriptions have been taking back up to ten dollars of illicit proceeds under the table*.

Foreign aid includes DRR and other vulnerability reduction measures. If the “dirty money” were invested in the communities where the money came from, perhaps there would be no need for foreign aid to initiate and support DRR.

**China**

**China’s corruption costs have been estimated as 8% of spending from 1996-2005 contributing “to China’s massive environmental degradation, deterioration in social services, and the rising costs of housing, health care, and education” (Pei, 2007, p. 5)^42^—“Even a relatively low-level official can amass an illicit fortune in tens of millions of yuan” (Pei, 2007, p. 2)^42^. The destruction of so many schools in the 2008 Sichuan earthquake, which killed 75,000 people including 900 children in one school, was strongly suspected to have resulted from corruption in construction (Lewis, 2008b)^69^.

The Chinese government states that it is battling corruption; for instance, “Chinese prosecutors investigated more than 240,000 embezzlement, bribery and other cases involving official corruption from 2003 to 2009”(Forsythe, 2010, online)^70^. Nonetheless, “corruption remains rife and is one of the most potent sources of public anger” (Branigan, 2011, online)^71^.

**Impoverishment 7: Siphoning of public money**

The director of Global Financial Integrity (GFI), Raymond Baker, has described the development of offshore financial havens as “the ugliest chapter in global economic affairs since slavery” (Shaxson, 2011, p. 158)^50^. Diversion and siphoning of public money into private bank accounts, wherever they are held, is an aspect of corruption (described above), drawing money away from potential DRR activities. Inclusive of development funding, money is stolen and sent to private, offshore tax havens before it can be spent for other purposes, which could have included the public good such as for vulnerability reduction.

The fine distinction made here between corruption and the siphoning of public money is between illicit expenditure and direct theft. Both are practiced for private gain. As a driver of vulnerability at national levels, the siphoning of public money has no rival to the enormity of geographical extent and size of the sums involved.

According to Dorling (2010: 101)^17^, there is a:

…trillion-dollar-a-year flow of illicit funds to rich nations using webs of financial trusts, nominee bank accounts, numerous methods to avoid tax, and simple mispricing…Traditional bribery and corruption within poor countries accounts for only 3% of this sum. Despite this, when corruption is considered it is almost always that kind of corruption and not the other 97%, the corruption of very rich Western bankers and businessmen (and a handful of businesswomen)…orchestrated from [London], from New York, and from a plethora of well-connected tax havens.

Approximately 50% of banking assets and 33% of foreign investment by multinational corporations are routed offshore through small island financial centres, estimated by the IMF as amounting overall to US$18 trillion, equivalent to one third of the world’s GDP (Shaxson, 2011)^50^. Providing secrecy, an escape from financial regulation and taxation elsewhere, not only small islands are involved. The United Kingdom has been identified by the IMF as an offshore jurisdiction (Shaxson, 2011; OECD, 2010)^50^ ^72^ with its government encouraging that status and further covert activity (Murphy, 2011)^73^.

Illicit outflows mean that governments have less money to spend on development and sustainability activities (Collier, 2008; Colvin, 2011)^74^ ^75^. For example, before it could be spent on infrastructure and general construction, millions of dollars of international aid have been sent from some African countries to private external bank accounts, often in the countries of the origin of the aid (Baker et al., 2008)^68^. Ndung’u (2007, p. 3)^76^ asks about Africa:

How many Harvards could be established in this continent to create the critical mass of world-class engineers and scientists, and intellectual leaders needed to advance Africa’s development, expand industrial output and enhance employment creation in the era of knowledge from the half a trillion US dollars stored by a handful of Africans in save [sic] havens?

Meanwhile, GFI (2011a)^49^ reports crime, corruption and trade mispricing—inclusive of kickbacks, bribes, embezzlement and other forms of official corruption—as leading to illicit outflows from developing countries of over US$1 trillion a year. Amongst countries with the highest cumulative illicit financial outflows between 2000 and 2008 were China at US$2.18 trillion, Russia at US$427 billion, Mexico at US$416 billon, Malaysia at US$291 billion, Venezuela at US$157 billion and Nigeria at US$130 billion (GFI, 2011a)^49^. Approximately US$854 billion is a conservative estimate of total illicit financial outflows from Africa, as it could possibly be “as high as US$1.8 trillion” (Shaxson, 2011, p. 157)^50^.

Ndikumana and Boyce (2008)^77^ examined capital sent from 40 African countries between 1970 and 2004. They conclude that sub-Saharan Africa is a “‘net creditor’ to the rest of the world” (p. 6) because “Real capital flight over the 35-year period amounted to about $420 billion (in 2004 dollars) for the 40 countries as a whole” (p. 6). That amounts to over double these countries’ combined external debt (Ndikumana and Boyce, 2008)^77^. The difference between the assets and the liabilities is that “private external assets belong to a narrow, relatively wealthy stratum of its population, while public external debts are borne by the people through their governments” (Ndikumana and Boyce, 2008, pp. 27-28; see also Ndikumana and Boyce, 2011)^77^ ^78^.

From 48 Least Developed Countries between 1990 and 2008, illicit flows—mainly to developed country banks and offshore financial centres—represented “a net resource transfer of about US$197 billion into the rest of the world (mainly developed countries)” (UNDP, 2011, p. 14)^79^. If such funds were applied appropriately internally, then development, poverty alleviation, and DRR could receive significant boosts through domestic initiatives.

**India**

**One estimate asserts that “rich Indians have spirited abroad the equivalent of half of India’s GDP over six decades” (Bidwai, 2010)^22^. Another states that the total illegal outflows of cash from India’s independence in 1948 until 2008 would add up to at least US$462 billion with compound interest (Kar, 2010)^47^. These sums may have been from private income sent overseas to avoid Indian taxation or siphoned from public funds. Either way, public money in India is being severely depleted by this activity. In January 2011, after an intervention from India’s Supreme Court regarding illicit flows ending up in bank accounts overseas, the Indian government took steps to try to curtail the problem, citing that they had detected tax evasion worth US$3.3 billion over the previous 18 months (Pasricha, 2011)^80^ .

**Kenya**

**In August 2007, Rice (2007, online)^81^ reported that (see also Githongo, 2005)^48^:

The breathtaking extent of corruption perpetrated by the family of the former Kenyan leader Daniel Arap Moi was exposed…in a secret report that laid bare a web of shell companies, secret trusts and frontmen that his entourage used to funnel hundreds of millions of pounds into nearly 30 countries including Britain. The 110-page report…alleges that relatives and associates of Mr Moi siphoned off more than £1bn of government money…The assets accumulated included multimillion pound properties in London, New York and South Africa, as well as a 10,000-hectare ranch in Australia and bank accounts containing hundreds of millions of pounds. The report, commissioned by the Kenyan government, was submitted in 2004, but never acted upon.

**Tajikistan**

**A report by the US ambassador to Tajikistan alleges that although the country experiences many disasters and is surrounded by difficult neighbours, the problems witnessed emerge internally. In particular, Tajikistan’s president, Emomali Rahmon (Harding, 2010, online)^46^:

runs the ex-Soviet republic's economy for his own personal profit: ‘From the president down to the policeman on the street, government is characterized by cronyism and corruption.’…Tajikistan’s sole industrial exports are aluminium and hydroelectricity. But most of the revenues from the ‘technically state-owned Tajik Aluminium Company (Talco) end up in a secretive offshore company controlled by the president…The state budget sees little of the income’.

Tajikistan has high disaster vulnerability and needs DRR, but funds that could be available disappear due to the leader’s corruption.

## Integrating DRR to avoid DRC

Why so many underdeveloped countries remain underdeveloped, or why the extent and levels of poverty and vulnerability prevail, are questions that cannot be answered without considering the colossal sums that accrue in private offshore bank accounts. Corruption is cited as an impediment to development (Collier, 2008, p. 66)^74^ and “the biggest threat to developing economies” (Colvin, 2011, online)^75^. These comments equally apply to the other behaviours presented above. To consider DRR should entail questioning these causes of persistent poverty and persistent vulnerability, in turn being the prime cause of disaster risk.

Notwithstanding similar recommendations made years earlier (e.g. Hamza and Zetter, 1998; Lewis, 1999)^10^ ^82^, recommendation 7.5 of the Global Assessment Report (UNISDR, 2009)^5^ encouragingly expresses that “a set of governance arrangements is needed for disaster risk reduction, poverty reduction and climate change adaptation, which is capable of ensuring that risk considerations are factored into all development investments” (p. 181).

That, as far as it goes, is how it should be. The evidence cited in this paper indicates that much more is at stake. As well as development, there is contra-development. Restriction of UNISDR’s (2009)^5^ recommendation 7.5 to only the three named sectors, and the suggestion that “development of a single governance framework for risk reduction would seem to offer opportunities for more effective policy implementation and for avoiding duplication and lack of coordination” (p. 181), indicate an approach too narrow for sufficient DRR when caused by the behaviours exemplified above.

UNDP (2010, p. 1)^83^ links vulnerability and disasters with governance:

Principles of good governance include broad participation, transparency, accountability, efficiency and responsiveness. All are as important for DRR as they are for development at large. A central criterion of good governance — namely, the principle of ensuring that the voices of the poorest and the most vulnerable are heard in decisions about the allocation of resources affecting them — is essential for effective DRR and sustainable disaster recovery.

UNDP (2010)^83^ speaks to democratically elected governments as the implied norm and, as with the UNISDR reports, assumes an epitome of the good. Without acknowledging the reality of the bad and the ugly—that is, of mal-governance, greed, and incompetence amongst others—this document also paints over the cracks in its façade. Why is it that “Many disaster-prone countries have not embraced mainstreaming DRR concerns into development practices as an underlying principle” (UNDP, 2010, p. 2)^83^? If it is shortages of money or political will, then the motives behind these superficial excuses have been amply demonstrated in this paper.

Documents produced by the United Nations are not the only sources of myopic visions of reality. Some academic documents also fail to go far enough. As one example, a paper jointly produced by 16 authors (Wisner et al., 2011)^84^, searches for incentives to achieve political will for the successful implementation of DRR. Corrupt practises offer incentives of personal profit of a magnitude far outweighing any, at a personal level, that might be achieved by DRR – and require less work for their installation. The paper makes limited active mention of the scale of corruption in all of its forms or of details regarding other illicit depletions of funds.

Perhaps the greatest failing of top-down institutionalised DRR is that it has been, and continues to be, separated from the reality of its contexts, whereas it is those contexts that actively contribute to the causes of disasters. By separating DRR from other separated sectors of government, the causes of people’s vulnerability to disasters must prevail (Lewis, 1999, pp. 5, 133, 134)^10^:

The pervasive conditions of vulnerability cannot be allocated as the responsibility of one desk or department; they are the prerogative of all desks and departments for all kinds of business and activity, both policy and practice...Institutional and organisational separation is the greatest single impediment to the integration of development and disaster reduction...Vulnerability has to be reduced by strategic processes within development; not by the ad hoc application of services that commence only after disaster has occurred, or with the identification of, and attendance to, already ‘vulnerable groups’.

Since these words were written, along with much similar discourse before and after, disasters of colossal magnitude have continued to tax disaster practitioners in their post-event focus whilst vulnerability creation has proceeded apace. The task for DRR is to adopt a strategy inclusive of vulnerability and its causative processes. Those processes exemplified in this paper should be made targets for primary attention, not only by development aid but also by curtailing financial outflows (Baker, 2012)^85^.

Psychological issues, prevailing in any government’s task of assessing risk for others (Lewis et al., 2011)^86^ , may have to be overcome. How to evaluate the science and to respond to it in parallel with political and economic priorities presents all governments with similar difficulties. Accepting that disasters happen, as disasters always have, is politically easier than professing to be able to prevent them and running the risk of backlash due to carrying the blame for failure.How much is the interpretation of science influenced by what is considered to be acceptable?

How much is science willing to rock the boat of governments? How much, also, are politicians of questionable ethics likely to want to change their expectations of positions of personal power?

Current focus on climate change adaptation, often at the expense of other DRR work, perhaps provides an opportunity to put the principles into practice to achieve ethical management. As massive amounts of money are directed towards climate change adaptation, irrespective of other DRR concerns, disciplined governance is required to ensure that funds are used in their entirety for the purpose for which they have been disbursed. The capital being (mis)appropriated by climate change work also includes intellectual capital, in terms of climate change knowledge focusing on their own internal knowledge-generating processes, and re-inventing what is known already, rather than embracing and building on wider sectors and learning from what other fields have long established.

DRR, too, should not be seeking to isolate itself. Moving beyond its presently perceived remit from the institutionalised perspective, DRR should be informed about, and prepared to deal with, all topics that have a bearing upon vulnerability. Otherwise, DRR will not succeed (Lewis, 2011a, p. 255)^87^:

Corruption acts as an engine of poverty and vulnerability… in poorer countries, where along with weak governance institutions there is often endemic corruption…Where corruption heightens community vulnerability, it exacerbates the need for adaptation measures. In regions plagued by weak governance, adaptation responses themselves may be particularly prone to corruption.

These interactions are shown in practice, when examining topics that are not always considered in DRR processes. For example, DRR should consider conflict, due to the inherent links between disaster risk and different levels of conflict. Corruption interacts with rebellion in complex ways, but can certainly feed grievances that lead to revolutions (Le Billon, 2003)^88^. Consequently, DRR must deal with corruption, conflict and their interactions.

As an example of interactions amongst numerous topics, a survey in Nairobi, Kenya (Shisanya and Kyayesi, 2007)^89^, found that respondents, in general, tended to be less concerned about flooding, El-Niño rainfall and “global warming” than about corruption, unemployment, crime, street children, garbage, transport and poverty. For DRR-related matters, pollution of the Nairobi River and HIV/AIDS (along with perceived immorality/promiscuity) did feature prominently. For some, to engage in DRR means engaging in topics concerning them immediately in tandem with demonstrating the links amongst those topics. That will link day-to-day known interests with longer term, more diffuse interests.

The prominence, but not exclusivity, of corruption is particularly notable. Bello et al. (2005)^90^, for instance, use the example of the Philippines to explain that the country’s problems and the resulting vulnerabilities would still exist even without corruption (p. 244):

While corruption definitely needs to be condemned, it is not the reason behind the country’s stagnation. A more adequate explanation lies in the state being subjugated by a succession of ruling elite factions to serve narrow interests instead of the larger goals of sustainable development and social justice.

Arguably, the second sentence perfectly defines a usual form of “corruption”. Rather than quibbling over definitions (see also the earlier discussion under “Impoverishment 7: Siphoning of public money”), Bello et al.’s (2005)90 point is well taken in exemplifying Table 1’s other three categories under “impoverishment”. DRR can happen despite corruption and can fail in corruption’s absence, but that does not mean that DRR and corruption are separable—as amply illustrated, for example, by the links between illegal logging and corruption (Goncalves et al., 2012)^91^ which, as demonstrated above for Pakistan then augments vulnerability.

Now that the G20 are committed to addressing illicit financial flows and have made explicit links between poverty and tax haven secrecy (GFI, 2011c)^92^, it is clear that DRR has for too long institutionally and conceptually separated itself from its social, economic and environmental contexts. DRR needs to integrate, not separate. Closer integration at global and international levels would hopefully encourage emulation at local levels. Sulston (2010, online)^93^ offers ways of thinking of which DRR should take note:

We certainly can survive but we can only do it by thinking in a rather larger and more collective way…The natural sciences will need to work in conjunction with the social sciences and governance if we are to ensure we will address challenges in an effective way. The challenges lie not so much in the natural sciences but in the social sciences and governance.

## Can bad and ugly become good?

Taking on these recommendations and better integrating DRR with other process does not in itself guarantee a disaster-free world—although DRR in itself has never aimed to guarantee a disaster-free world. Risks will always exist and decision-makers, necessarily, must always balance varying residual risks. It is important to acknowledge those residual risks, especially to determine if disasters could nonetheless be avoided through targeted interventions that do not, in turn, cause further risk-related (or other) problems.

What does this mean practically to achieve DRR in the face of DRC? Could a conceptual model be developed to guide vulnerable people and communities, along with higher-scale authorities and influences, to recognise, acknowledge and manage residual risks that remain after DRR has been integrated as much as feasible? Could such a conceptual model map out what needs to be done beyond fighting corruption or contra-development?

To a large degree, these conceptual models have long existed (e.g. Lewis, 1984; O’Keefe et al., 1976)^7^ ^21^ and continue today to be reiterated, refined and interpreted from different angles (e.g. Lewis et al., 2011; Wisner et al., 2012)^19^ ^86^. They frequently converge on the long-term, fundamental topics exemplified in this paper, especially with regards to changing culture and reallocating existing funds from processes that impede development—recognising endangerment and impoverishment as active, basic drivers of vulnerability and disaster risk.

The practice from these conceptual discussions appears through examples of successful DRR and development. Certainly, DRR being established and advancing is an overall “good”, but in many instances that has happened largely regardless of vulnerability. Similarly, recognising and admitting some of the drivers of vulnerability is also a “good”. But recognition, however important, is only the first step. Massively “bad” and “ugly” drivers persist in practice as a pernicious and prevailing reality, triggered by negative aspects of human behaviour. If DRR is being accepted as or aimed to be a “culture”, “contra-culture” exists as does “contra-development”. At the moment, the contra-culture dominates, as shown by this paper’s examples.

Ways of working for DRR are required so that internationally conceived and jointly contributed official documents – and some academic texts – begin to absorb and respond to the realities. A first step would be recognising courageously committed investigative reporting that lays out and details the large scale of “the bad and the ugly”. Without tackling all vulnerability drivers – that are the roots of DRC – the conditions of DRC will continue to prevail over attempts at DRR.
